# Wide-Field-of-View Multispectral Camera Design for Continuous Turfgrass Monitoring

**DOI:** 10.3390/s23052470

**Published:** 2023-02-23

**Authors:** Lien Smeesters, Jef Verbaenen, Luca Schifano, Michael Vervaeke, Hugo Thienpont, Giancarlo Teti, Alessio Forconi, Filippo Lulli

**Affiliations:** 1 Brussels Photonics (B-PHOT) and Flanders Make, Department of Applied Physics and Photonics, Vrije Universiteit Brussel, Pleinlaan 2, 1050 Brussels, Belgium; 2 Robotech, Via Mazzini 82, 19038 Sarzana, SP, Italy; 3 Turf Europe, Via Malasoma 24, 56122 Pisa, PI, Italy

**Keywords:** turfgrass monitoring, vegetation indices, wide field of view, camera design, multispectral camera, thermal imaging, modulation transfer function, demonstrator setup

## Abstract

Sustainably using resources, while reducing the use of chemicals, is of major importance in agriculture, including turfgrass monitoring. Today, crop monitoring often uses camera-based drone sensing, offering an accurate evaluation but typically requiring a technical operator. To enable autonomous and continuous monitoring, we propose a novel five-channel multispectral camera design suitable for integrating it inside lighting fixtures and enabling the sensing of a multitude of vegetation indices by covering visible, near-infrared and thermal wavelength bands. To limit the number of cameras, and in contrast to the drone-sensing systems that show a small field of view, a novel wide-field-of-view imaging design is proposed, featuring a field of view exceeding 164°. This paper presents the development of the five-channel wide-field-of-view imaging design, starting from the optimization of the design parameters and moving toward a demonstrator setup and optical characterization. All imaging channels show an excellent image quality, indicated by an MTF exceeding 0.5 at a spatial frequency of 72 lp/mm for the visible and near-infrared imaging designs and 27 lp/mm for the thermal channel. Consequently, we believe our novel five-channel imaging design paves the way toward autonomous crop monitoring while optimizing resource usage.

## 1. Introduction

Sustainable precision farming is of utmost importance for modern agriculture, mapping important soil and plant properties to obtain reliable environmental management by pursuing resource optimization, including the efficient application of water, fertilizers and pesticides. Irrigation management is becoming increasingly important and challenging, with view to how climate change is inducing higher summer temperatures and prolonged droughts. As agriculture is responsible for 24% of water abstraction in Europe, an optimized irrigation schema is undeniably required to achieve more-efficient water use [1]. The extensive use of pesticides should be avoided because they can contaminate the soil, water and vegetation and are toxic to animals. The European Commission aims to reduce the use of chemical pesticides by 50% by 2030 while promoting environmentally friendly pest control alternatives [2].

Precision agriculture is driving new developments for crop and environmental monitoring. A wide range of crop-monitoring camera systems are currently already available, such as the ones of MAPIR, MicaSense and Sentera, offering multispectral sensing systems and providing information on vegetational indices [3]. Successful results have been demonstrated for crop and turfgrass monitoring, including the assessment of the vegetation coverage, the sensing of drought stress and the monitoring of the normalized difference vegetation index (NDVI) [4,5,6,7]. These camera systems are, however, typically optimized for use on uncrewed aerial vehicles (UAVs), featuring a limited field of view (FOV), generally smaller than 50°, and hence, they are unsuitable for the fixed proximity monitoring of agricultural and green area surfaces. The main advantages of UAVs provide a high flexibility, a high spatial resolution, and a nondestructive field evaluation. However, its main disadvantages include still having a high cost while also being quite complex from an operational point of view.

**Figure 1:**
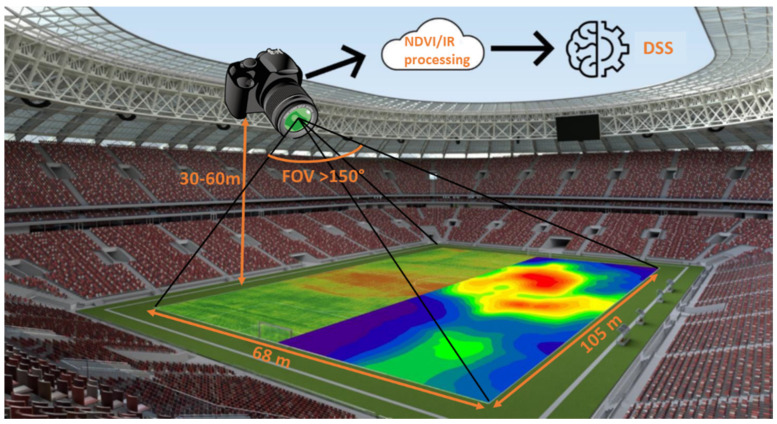
Targeted novel camera design, using fixed camera positioning while observing the whole field thanks to the wide field of view. The multichannel camera design enables the calculation of the vegetational indices, providing input to a decision-support system (DSS).

Our research aims for the development of a novel wide-field-of-view multichannel camera design, offering real-time and continuous multispectral measurements combining visual and thermal image data, allowing the early detection of water stress, disease and pest pressure. Particularly, a five-channel camera design has been investigated, combining four visible and near-infrared (NIR) spectral channels with a single thermal imaging channel, each of which has a similar field of view but is spectrally optimized to give greenkeepers insights into the health and needs of the field, enabling preventive and eco-friendly management. As a case study, we focus on the environmental monitoring of recreational turfgrass areas, including sport stadia, golf courses and horse racing, which require high-quality fields. We aim for autonomous and continuous monitoring where the camera system is fixed on the lighting poles, enabling the identification of turf stress in its early stage (Figure 1). This fixed positioning enables an earlier detection than the conventional field-scanning designs using UAVs and cameras mounted on, e.g., tractors and grass-mowing devices, while excluding the need for an operator. However, the main difficulty is that the optical design requires a larger field of view to maximize the observation area, limiting the number of required camera units, thus minimizing the cost.

Wide-field-of-view (WFOV) camera systems impose stronger design challenges than their small FOV counterparts thanks to their shorter focal lengths and therefore their small f-numbers [8]. Additionally, off-axis aberrations might show a larger influence on the design performance. Thanks to refractive lens designs, the largest FOV, up to a full FOV exceeding 200°, can be achieved by using fisheye-lens designs. Optical designs with a full FOV between 100° and 200° can be achieved by using topogon, hologon and retrofocus designs. A technology scouting and a literature review were performed to find WFOV visible, near-infrared and thermal camera designs. In general, WFOV visible camera designs are more widely available than the thermal ones. Given that the camera designs are optimized for use within the visible wavelength range, a wide variety of designs has been developed and demonstrated, including commercial off-the-shelf (COTS) cameras (Table 1) and research-grade designs (Table 2). In general, these imaging designs contain a combination of different spherical and/or aspherical lenses. The research-grade aspherical designs tend to optimize toward the system dimensions and toward a reduction in the number of lens elements, giving rise to higher-order aspherical coefficients within the design. In contrast to the visible camera designs, thermal WFOV lenses are only limited reported at present (Table 3), while infrared camera-related technology is still a trending research topic [9]. To the best of our knowledge, no commercial off-the-shelf thermal camera system is available with a WFOV fisheye lens. The main suppliers offer only WFOV solutions up to field angles of 80°. Thanks to research-grade designs, a wider FOV has been reported, but it requires custom aspherical lenses.

We investigate the development and assembly of a cost-efficient five-channel design, optimized and dedicated for turfgrass monitoring. This includes the selection of the most suitable optical filters, the optimization of the imaging lenses and sensors and its assembly in a compact unit. During this development and component study, the state-of-the-art camera designs were used as benchmarks and enabled the selection of the most suitable components. Furthermore, a material optimization was performed because each wavelength range features its own optimally suited materials and sensors, enhancing the transmittance and sensitivity and thus also the optical performance [10].

This paper presents our novel wide-field-of-view multispectral camera design, featuring five imaging channels covering the visible, near-infrared and thermal wavelength bands, optimized for measuring vegetation indices. Section 2 presents the materials and methods and an overview of the multispectral camera design. Section 3 focuses on the camera assembly and proof-of-concept laboratory demonstrator setup, evaluating the FOV and image quality. Section 4 discusses the obtained results and the further integration of the design into an online maintenance control system. Finally, the conclusions are provided in Section 5.

**Table 1:** State-of-the-art COTS WFOV visible and near-infrared imaging designs.

Design Type	Number of Lenses	Effective Focal Length (mm)	f- Number	Full FOV (Degree)	Total Optical Length (mm)	Full Field f−θ Distortion (%)	MTF Value at a Frequency (lp/mm)	Detector Format (inch)	Surface Types	References
Fujifilm-86	-	2.7	1.8–16	136.3	>60.5	0.53	-	1/2	All SPH	[11]
Fujifilm-57	-	1.8	1.4–16	185.0	>60.3	0.8	-	1/2	All SPH	[11]
SunexDSL180	-	0.97	2.0	200	17.1	14	-	1/2.8	All SPH	[12,13]
Entaniya 220	10	-	2.0	220	36	2	>0.4 @ 100	-	All SPH	[13,14]
Nippon 1964	9	8	≥2.8	≥180	88	3.84	-	1.112	All SPH	[15]
Nippon 1971	12	6.3	≥2.8	220	205.9	3.14	-	1.112	All SPH	[15]
Olympus 1973	10	8	≥2.8	≥180	125	9.75	-	1.112	All SPH	[15]
Asahi 1985	10	8	≥2.8	≥180	139.7	0.02	-	1.112	All SPH	[15]
Coastal 1997	11	7.45	2.8	185	174	1.85	-	1.112	All SPH	[15]

**Table 2:** State-of-the-art research WFOV visible and near-infrared imaging designs.

Design Type	Number of Lenses	Effective Focal Length (mm)	f- Number	Full FOV (degree)	Total Optical Length (mm)	Full Field f−θ Distortion (%)	MTF Value at a Frequency (lp/mm)	Detector Format (inch)	Surface Types	References
Canopy lens	13	2.21	2.97	180	70.36	32.5	>0.6 @ 153	1/2	All SPH	[16]
Compact lens	6		2.5	230	17.3	<0.03	>0.3 @ 45	1/4	4 ASPH	[17]
Low cost	4	2.5	3	100	12.9	>40	>0.3 @ 40	1/4	2 ASPH	[18]
Simplified fisheye	6	1	2.8	160	11.6	0.15	>0.3 @ 115	1/3.2	1 ASPH	[19]
Ultrawide	7	1.1	2.4	160	25	<5	>0.4 @ 178	1/6	4 ASPH	[20]
Miniature	5	2.06	4	200	14.7	<4.5	>0.4 @ 75	2/3	1 ASPH	[21]
Zoom lens	11	9.2	2.8	180	140	10	>0.4 @ 50	1.112	2 ASPH	[22]
360 lens	7	1.43	2.0	190	27.0	<7.1	>0.1 @ 370	1/2.3	4 ASPH	[23]
Tracking lens	4	0.963	2.5	180	11.8	<1	>0.6 @ 59.5	1/3	1 ASPH	[13]
Fingerprint	3	-	1.6	128	<2.4	<1	≥0.5 @ 70	-	All ASPH	[24]
Spaceborne	5	3.3	2.9	140	85.5	74.6	≥0.4 @ 78	1/5.2	2 ASPH	[25]
Cascade	6	50	3.1	116.4	230.32	<0.2	≥0.285 @ 270	1/1.7	2 ASPH	[26]
Light field	4	17.5	-	60	-	-	-	1/1.8	SPH	[27]
Freeform	5	1.8	2.44	109	3.19	<2	≥0.31 @ 120	1/4.4	2 freeform	[28]

**Table 3:** Overview of the state-of-the-art thermal imaging designs.

Design Type	Number of Lenses	Effective Focal Length (mm)	Wavelength Range (µm)	f- Number	Full FOV (Degree)	Total Optical Length (mm)	Full Field f−θ Distortion (%)	MTF Value at a Frequency (lp/mm)	Surface Types	References
Commercially available WFOV visible imaging systems										
Fluke WFOV	-	-	8–14	-	46 × 34	165	-	-	-	[29]
FLIR wide-angle lens	-	9.66	8–14	1.3	45 × 33.8	38	-	-	-	[30]
Fotric wide-angle lens	-	-	8–14	-	76 × 57	-	-	-	-	[31]
Research on WFOV visible imaging systems										
Low-cost thermal lens	2	13.6	8–14	1.1	48	32.6	<3	≥0.5 @ 20	All ASPH	[32]
LWIR earth sensor	4	4.177	14–16	0.8	180	-	<0.25	≥0.5 @ 15	All ASPH	[33]
Spaceborne lens	3	-	8–14	1	140	86.12	<18.85	≥0.5 @ 15	1 ASPH	[34]
Monocentric design	2	63.5	3–5	1	100	150	-	≥0.45 @ 70	SPH	[35]

## 2. Materials and Methods: Optical Design Parameters and Trade-Offs

The development of the five-channel multispectral camera system first required an evaluation of the design parameters, including an optimal sensor selection and the optimization of the wavelength bands for turfgrass monitoring while pursuing a WFOV, enabling the mounting on the lighting poles in sport stadia. We first give a general overview of the design constraints and parameters, after which the outlook of the visible and NIR and thermal camera channels are discussed in detail, in the following subsections.

Thanks to the mounting of the camera system, we can aim for a flexible integration by using fixed lenses, thus avoiding mechanical movements, at a height between 30 and 60 m, requiring a large depth variation and a correspondingly wide depth of field. Next, we aim to capture the entire sports stadium at once by using a single camera. The standard size of a sports stadium, as defined by FIFA [36], is set at 105 m × 68 m, imposing a minimal required full FOV of 120° × 97°. This FOV would impose that the camera always be positioned in the center of the sport field. To extend the range of mounting possibilities while extending the application to other recreational fields and golf courts, we aim for a minimal full FOV of 150° for all imaging channels. Larger FOV values are preferred, pointing toward the direction of fisheye lenses, which enable the largest FOV, that of 180° [8,37].

Insights into the camera design parameters was gained on the basis of using back-of-the-envelope calculations. The number of pixels, the sensor size and the lens focal length depend on the FOV, the resolution, and the working distance [38]. The required number of pixels is calculated by using Equation (1), where the FOV is expressed in mm, which is the area under inspection that the camera needs to acquire, and the resolution is defined as the size of the smallest feature that one wants to detect in the image. Following the Nyquist theorem, 2 pixels per smallest feature are included. The sensor and lens materials were selected to match with the different wavelength ranges, and they are a silicon sensor for the visible wavelength range and a microbolometer for the thermal range. For the dimensions of the image sensor, it is preferred that the format of the lens be larger than or equal to the sensor size. In the case the lens would be smaller, the image experiences vignetting, causing regions in the sensor outside of the lens area to be black. The focal length (f) of the lens can subsequently be calculated by using Equation (2). In general, smaller focal lengths indicate a more difficult design and one that is more susceptible to aberrations. An optimal image quality was pursued. However, no special care was taken to account for distortion, because this aberration can be corrected in postprocessing, typically by using a distortion model [39,40].

(1) $$\#\;pixels=\frac{2\times FOV\;\left(\mathrm{mm}\right)}{resolution\;\left(\mathrm{mm}\right)}$$

(2) $$f\left(\mathrm{mm}\right)=\frac{\#pixels\times pixel\;pitch\;\left(\mathrm{mm}\right)\times working\;distance\;\left(\mathrm{mm}\right)}{FOV\;\left(\mathrm{mm}\right)}$$

**Figure 2:**
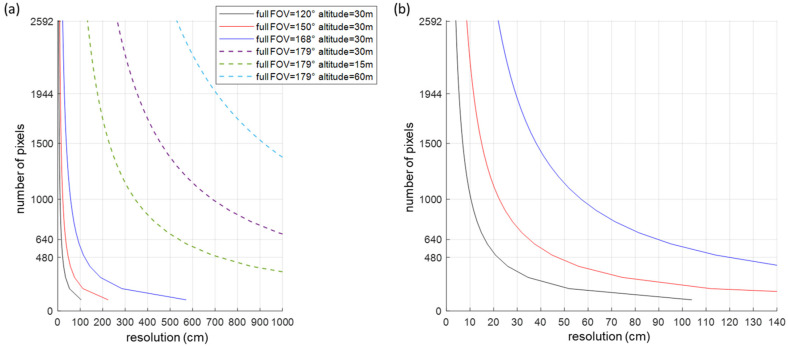
Required number of pixels as function of the resolution, for different FOV and working distances: (**a**) a full FOV from 120° up to 179°; (**b**) a closeup of (**a**) focusing on the small resolution values between 0 cm and 140 cm, for full FOV of 120°, 150° and 168°. A steep increase of the number of pixels can be observed with improved resolution.

**Figure 3:**
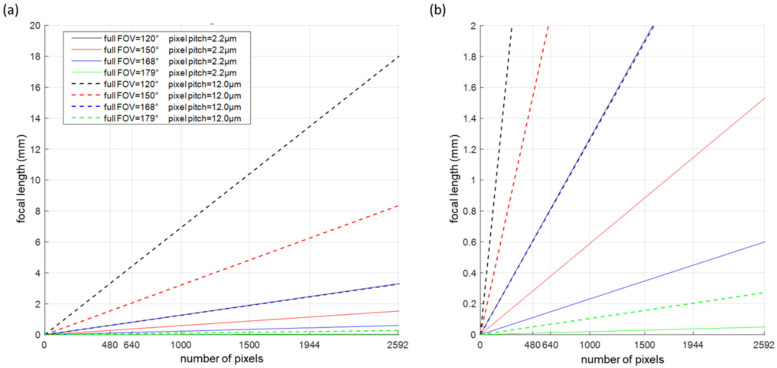
Lens focal length as function of the number of pixels, when using a working distance of 30 m: (**a**) a full FOV from 120° up to 179° and a pixel pitch of 2.2 µm and 12 µm; (**b**) a closeup of (**a**) but at up to 5292 pixels. In general, a linear scaling of the design can be observed.

By using the above equations, the required number of pixels is studied as function of the image resolution, for different FOVs and working distances (height of the camera). A steep increase in the required number of pixels is visible for decreasing resolution values, thus improving resolution while also increasing with the FOV (Figure 2). A resolution of 40 cm requires at least 300 pixels for a FOV of 120°, 600 pixels for a FOV of 150° and 1400 pixels for a FOV of 168°. For a FOV of 150°, a resolution of 20 cm can be achieved by using 2000 pixels. The number of pixels and the FOV influence the lens design and specifically its required focal length (Figure 3). With regard to 640 pixels with a pixel pitch of 2.2 µm, focal lengths less than 1 mm are required for FOVs exceeding 120 degrees, and this increases to 1–5 mm when enlarging the pixel pitch to 12 µm. Typically, thermal sensors feature larger pixel sizes than visible ones do, enabling the inclusion of lenses with larger focal lengths but often resulting in a reduction in the number of pixels that negatively impact the resolution. Nevertheless, a trade-off between the number of pixels and pixel pitch, influencing the cost of the camera, the FOV and imaging resolution, needs to accepted during the camera development.

To gain insights into the turfgrass parameters and its spectral reflectance, a multispectral imaging design is required. Today, different methods have been exploited to enable multispectral imaging, including single-sensor and multisensor layouts. A single-sensor layout can be realized by using, among others, a multispectral filter array, a filtered lenslet array and diffractive optics, while a multisensor design can contain different entrance apertures for each of the channels or contain a beam-splitting component [41,42,43]. We decided to work with a multisensor camera layout, maximizing the achievable resolution while avoiding the need for multispectral demosaicing.

For each of the multispectral imaging channels, the selected wavelength ranges are optimized toward the turfgrass spectral reflectance. More specifically, the spectral reflectance of turfgrass has been shown to be sensitive to agronomical and biological parameters, including color, chlorophyll, carotenoids, nitrogen content, fertilizer and herbicide application rates, irrigation amount and uniformity, and management practices such as mowing height [44,45]. The sensing of different visible and near-infrared vegetation indices was envisioned, defined as crop evaluation numbers obtained by combining different reflection bands. Given its canopy density and structure, several vegetational indices can be used to accurately monitor and diagnose turfgrass surfaces, with the capability of discriminating color, species, varieties, and sward status [46]. Among others, the NDVI, which considers the red and near-infrared reflectance, is widely used as a reflectance-based stress indicator and correlates with turfgrass quality, which is itself a combined function of density, uniformity, color, nutritional status and water status. We pursue an optimization of the spectral bands toward the optical properties of turfgrass while covering a multitude of vegetation indices.

Thermal imaging exploits the fact that everything above 0 Kelvin emits radiation and that this radiation is affected by plant diseases and pathogens and by a plant’s water status [4,47,48]. Therefore, thermal images provide valuable information on irrigation needs. In particular, and to approximate water stress with thermal data, the crop water stress index (CWSI) has been proven to be an important parameter. During periods of water stress, the crop limits its transpiration by using stomata regulation, increasing plant temperature. For sports stadia, for example, the thermal images will also provide information on the underground heating system, easing the detection of defects.

On the basis of the above design parameters evaluation, a design optimization was performed while pursuing a compact and cost-efficient system. The optimized layout is discussed in the following subsections.

### 2.1. Visible and Near-Infrared Camera Design

The visible and near-infrared imaging channels each combine a fixed fisheye lens (Lensation BF5M15828S125) with a CMOS sensor (MT9PO31 sensor of ON Semiconductor). The fisheye lens has an aperture of 2.8 mm, a focal length of 1.58 mm and a back focal length of 5.65 mm while featuring a FOV of 180°. The camera sensor has a sensor size of 5.7 mm × 4.3 mm, featuring 2592 × 1944 pixels, each with a pixel size of 2.2 µm × 2.2 µm.

Each of the 4 imaging channels is supplemented with a different bandpass filter, enabling the detection of different spectral ranges, where the selected wavelength bands are based on previous studies covering the spectral signatures of turfgrass species, in combination with a technology that scouts for the available optical filters. Specifically, we considered the following 4 wavelength bands: (1) 680–720 nm (MidOpt BP695), (2) 580–610 nm (MidOpt BN595), (3) 645–675 nm + 750–950 nm (MidOpt DB660/850) and (4) 405 nm–690 nm (MidOpt BP550). The wavelength ranges are carefully selected to enable the calculation of the main important vegetation indices: the dark-green color index, the chlorophyll indices, the NDVI, the simple ratio index (SR), and RedEdge [45,46]. To avoid vignetting, the filters need to be positioned between the last lens surface and the image sensor. Therefore, the dimensions of the filters were fine-tuned to a diameter of 10 mm and a thickness of 1 mm, to allow cementing on the lens mount.

### 2.2. Thermal Camera Design

The thermal imaging design was optimized for a spectral range from 8 µm to 14 µm because this wavelength range has been shown to be optimal for water monitoring and health status sensing [47,48]. Furthermore, within this wavelength range, the solar radiation is negligible, and the atmosphere is showing only a small absorptance. The measurement of the radiation emitted by the Earth’s surface in this spectral range and the knowledge of the emissivity of the surface (typically between 0.95 and 0.99 for vegetation cover) allows for estimating the surface temperature of vegetation. The difference between the surface temperature of the canopy and the air temperature provides a good indication of the water status of the plant.

The thermal camera is assembled by combining the thermal image sensor (TE-EV2 of i3Systems (640 × 480 pixels, 12 µm pixel pitch)) with the fisheye thermal lens (SupIR 2.6 mm f/1.4 of Ophir Infrared Optics). The detector is an uncooled microbolometer enabling scene range temperatures from −10 °C to 150 °C. The thermal lens features a WFOV of 168.8° when combined with a 640 × 480 sensor format while showing a high transmittance of 96% within the thermal wavelength range. It is a fixed athermalized lens with a depth of field from 0.25 m to infinity, a focal length of 2.6 mm, a f-number of 1.4 and a back focal length of 8.03 mm.

## 3. Results: Proof-of-Concept Demonstrator Evaluation

**Figure 4:**
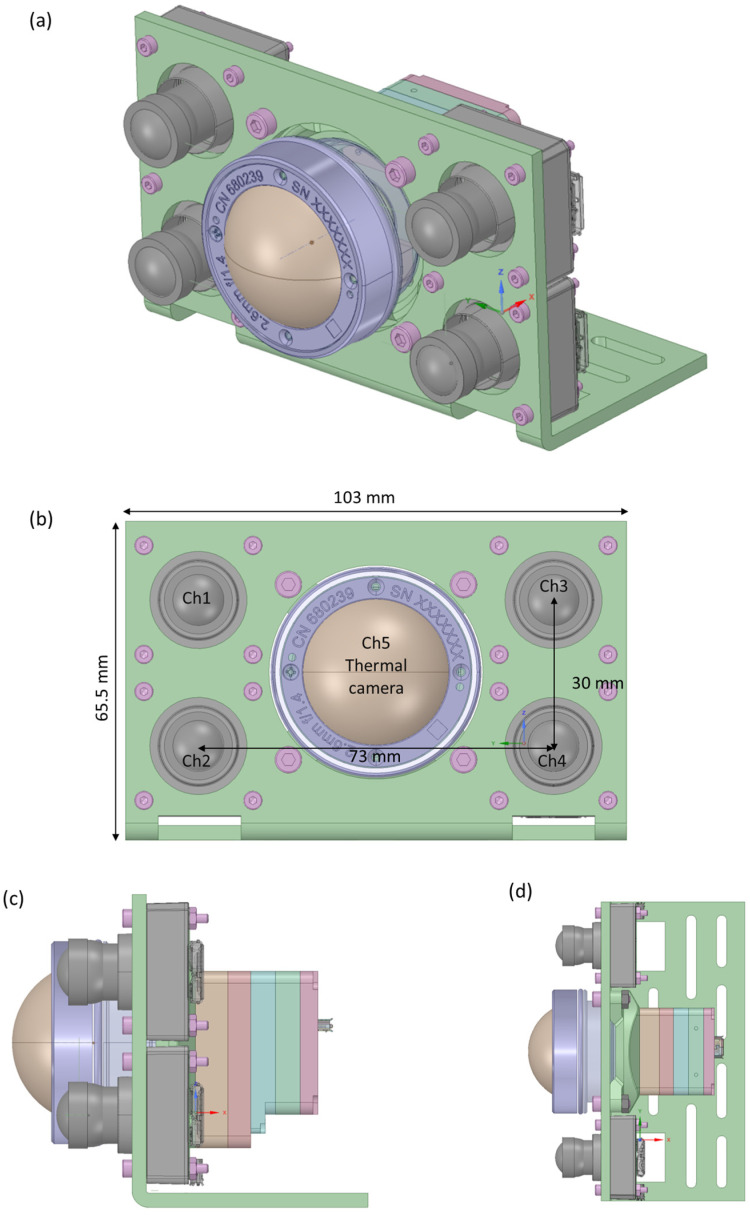
Layout of the 5-channel camera design: (**a**) 3D view, (**b**) front view indicating the position of the thermal camera in the center surrounded by the visible and NIR imaging channels, (**c**) side view indicating the mounting of the imaging sensors, (**d**) top view.

The five imaging channels were integrated into a multispectral camera layout by using a custom mounting plate (Figure 4). The mechanical design was developed in Ansys SpaceClaim, 3D CAD-modeling software. The thermal imaging channel is positioned in the center, surrounded by the four visible and NIR channels, and the total layout has a compact dimension of 65.5 mm × 103 mm. Considering the visible and NIR channels, the CMOS sensor was positioned in a holder with S-mount, enabling to screw-on the fisheye lenses in a holder with S-mount, enabling the fisheye lenses to screw on. The thermal imaging channel required a custom connector to position the thermal lens in front of the thermal sensor. For each channel, the distance between the lens and the image sensor is carefully set and fine-tuned toward the optimal image quality. All channels feature a USB interface.

**Figure 5:**
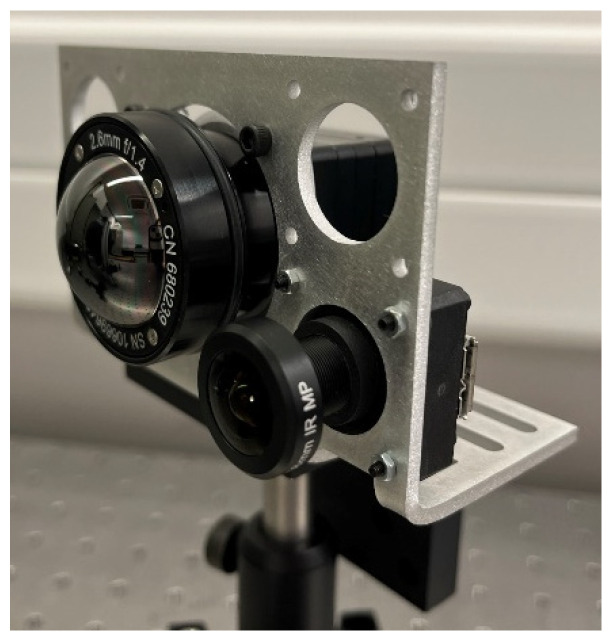
Laboratory test setup featuring the thermal channel and one of the visible and NIR imaging channels.

A laboratory characterization of the visible and NIR and thermal imaging systems was performed (Figure 5). First, the operation of the filters was accurately determined by using a broadband transmission spectroscopy setup (400–1000 nm), after which the image quality was characterized by imaging a slanted edge and by subsequently calculating the modulation transfer function (MTF). The slanted-edge technique requires only one snapshot of the target to determine the MTF at all frequencies. Particularly, the transition from the dark side to the bright side of the edge gives an indication of the aberrations and/or diffraction related to the properties of the imaging system while allowing the calculation of the MTF as the normalized Fourier transform of the first derivative of the edge spread function. The MTF calculation is performed using the ImageJ plugin called the slanted-edge modulation transfer function (SE_MTF) [49,50].

### 3.1. Characterization of the Visible and Near-Infrared Camera Channels

**Figure 6:**
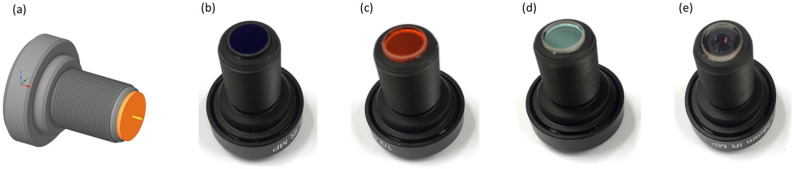
Cemented optical filters on the wide FOV lens: (**a**) technical drawing indicating the cementing of the filters, with the optical bandpass filter indicated in orange; (**b**) channel 1 featuring the MidOpt BP695 filter, (**c**) channel 2 featuring the MidOpt BN595 filter, (**d**) channel 3 featuring the MidOpt DB660/850 filter, (**e**) channel 4 featuring the MidOpt BP550 filter.

**Figure 7:**
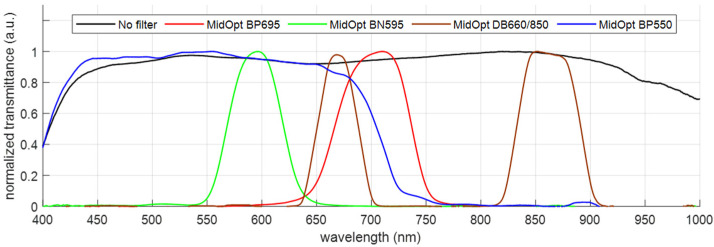
Transmittance spectrum for the different visible and NIR imaging channels, after the cementing of their respective optical filters.

Each of the visible and near-infrared camera channels features a different optical bandpass filter, cemented in front of the image sensor (Figure 6). For each channel, the spectral transmittance was measured after cementing the filters, by using a broadband halogen illumination source, emitting light within the 400–2500 nm spectral range, and a broadband spectrometer (Avantes AvaSpec3684), enabling the measurement of the transmittance spectrum between 400 nm and 1000 nm, with a resolution of 1.4 nm. The different transmission bands indicate a high transmittance in the respective pursued wavelength regions (Figure 7). With the transmittance at 1/e^2^, the following spectral bands are measured: (1) 648–750 nm when using the MidOpt BP695 filter, (2) 556–634 nm when using the MidOpt BN595 filter, (3) 641–697 nm + 823–900 nm when using the MidOpt DB660/850 filter and (4) 400–723 nm when using the MidOpt BP550 filter. As a reference, the transmittance of the lens before cementing the filters is also included, indicating a broadband transmission within the 400–1000 nm wavelength range, visualizing the spectral influence of the different optical filters.

**Figure 8:**
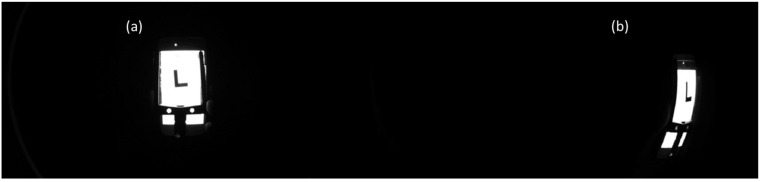
Image of the slanted edge evaluating the image quality of the visible and NIR camera channels: (**a**) on-axis positioning, (**b**) off-axis positioning (at 179°), indicating the presence of barrel distortion.

**Figure 9:**
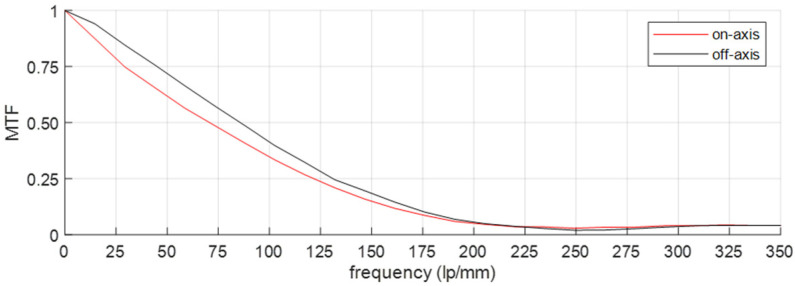
MTF measurement of the visible and NIR camera channels, showing on-axis and off-axis (at 179°) positioning of the object, indicating good image quality.

The FOV and the image resolution are evaluated by imaging a slanted-edge test chart (Thorlabs R2L2S2P Slant Edge MTF target). The target chart was moved along the FOV, enabling on-axis and off-axis evaluations (Figure 8). Experimentally, a FOV of 179° was observed. Each of the four channels gives the same image quality, independent of the bandpass filter. A slight difference in the MTF was measured for the on-axis and off-axis positionings (at 179°); however, in general, good image quality was observed (Figure 9). More specifically, an MTF value ≥ 0.5 was observed at 72 lp/mm, while we obtained a value of 87 lp/mm and 109 lp/mm for an MTF value ≥ 0.4 and ≥0.3, respectively. These values compete to be state of the art. Our design clearly surpasses the “compact lens”, “low-cost lens”, “miniature lens”, “zoom lens”, “fingerprint lens” and “spaceborne lens” that are presented in Table 2. The “canopy lens”, “ultrawide lens” and “cascade lens” exceed our performance values, but they use ≥6 lenses featuring a large number of aspherical coefficients.

Barrel distortion can be identified as the main present aberration because it is clearly visible in the images. This aberration originates from the differences in the transverse magnification that each of the off-axis image points face, implying that the image points are observed inward from their ideal positioning. This aberration is typical of WFOV designs, and different software algorithms are already available to correct this aberration during postprocessing [39,40].

### 3.2. Characterization of the Thermal Camera Channel

**Figure 10:**
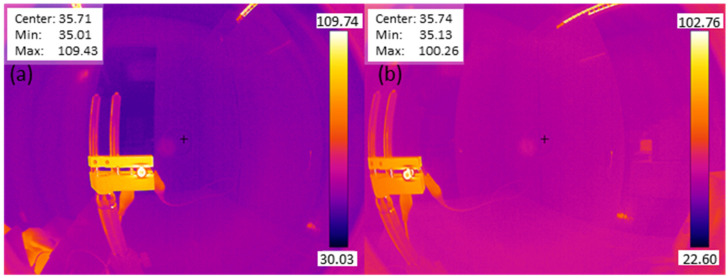
Thermal image of a mounted LED, heating up a metal plate: (**a**) on-axis positioning, (**b**) off-axis (at 164°) positioning.

The thermal camera design is characterized by evaluating the FOV and the MTF. The FOV is measured by positioning a high-emitting (heated) LED within the object plane and subsequently moving its position along the FOV by using translation stages (Figure 10). The measured full FOV equals to 164°.

**Figure 11:**
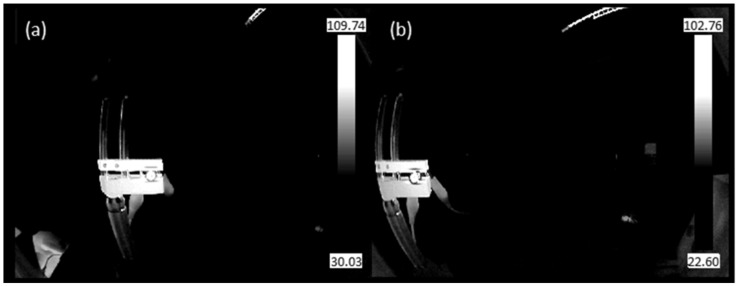
Grayscale thermal image of a mounted LED, heating up a metal plate after background correction: (**a**) on-axis positioning, (**b**) off-axis positioning (at 164°). These images were used as inputs for the MTF calculation.

The MTF measurement uses a slanted-edge approach, similar to that for the visible and NIR imaging channels. However, one key difference is that the traditional slanted-edge test chart cannot be used, because the thermal camera does not account for the visible NIR spectrum but instead measures thermal radiation and thus temperatures. An image of the slanted-edge target would result only in an image of the target with a certain color, corresponding to a certain temperature. Therefore, the slanted edge is artificially created by contrasting a low temperate with a high temperature, and this contrast accounts for room temperature and a heated metal plate, respectively, where the latter one is heated by using a high-power LED. The temperature contrast is clearly visible in the color-scale images, as typically achieved from thermal image sensors (Figure 10). To obtain the black-white edge for the MTF calculation (Figure 11), the achieved image is converted to grayscale, and a background correction was applied. The background correction is obtained by sequentially applying the following three steps: (1) draw rectangle in the surrounding environment, ensuring no object is present in the background; (2) calculate the minimum in the rectangle; and (3) subtract the background value from the image. The resulting images, as depicted in Figure 11, serves as the basis for the MTF calculation, where the contrast between the present black-white edge is considered as the slanted edge.

**Figure 12:**
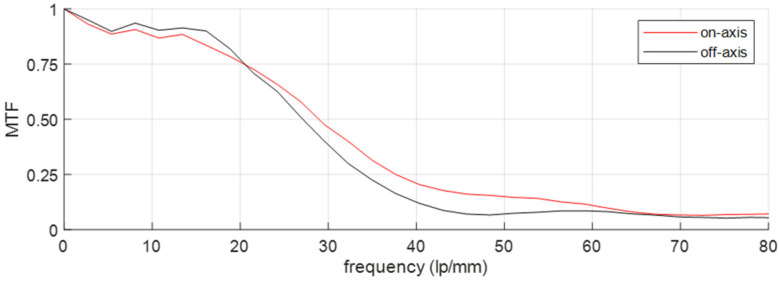
Measured MTF for the thermal camera channel, for the on-axis and off-axis (at 164°) configurations, surpassing the current state of the art.

The calculated MTF indicates good image quality (Figure 12). An MTF value of 0.5 is achieved for a frequency ≥ 27 lp/mm, exceeding the current state of the art, which is presented in Table 3. Only the “monocentric design” exceeds the specifications; however, this design does not cover the full thermal wavelength band. Consequently, the excellent image quality, indicated by the MTF, in combination with the compact design, enables the boosting of the current thermal camera performance. The main aberration of our thermal camera design is the barrel distortion, similar to the main aberration of the visible and NIR imaging channels, but this can be corrected in postprocessing. Given the practical implementation, a radiometric calibration will be performed on the field, including cold and hot targets.

## 4. Discussion and Future Perspectives

Precision farming is of utmost importance to support greenkeepers in the sustainable management of their crops. Current precision farming tools can be improved by optimizing sensor design and by adding reliable machine-learning algorithms to the image processing.

In this paper, we present an optimized five-channel camera system, providing a multiparameter sensing design. Our multichannel design enables a broadband spectral measurement, covering visible, NIR and thermal wavelengths while offering a WFOV (Table 4). Current state-of-the-art multispectral camera designs typically show a limited FOV, suitable for integration with UAVs. In contrast, we targeted a camera design suitable for a fixed mounting in, e.g., lighting fixtures, enabling continuous monitoring and early detection.

A comparison of the design specifications of the visible and NIR camera channels (Table 4) with the state of the art (Table 2) shows that the f-number of our proposed camera channels is within the range of the typically reported f-numbers: between 1.6 and 4.0. The f-number of our design equals the one of the “simplified fisheye” and “zoom lens”. Like the “simplified fisheye” design, our proposed system features a larger FOV, a comparable MTF and a larger total optical length. With respect to the “zoom lens”, our design shows a comparable FOV, a shorter optical length and a better MTF. The “compact lens” and “miniature lens” exceed our specified FOV but show a lower MTF.

A comparison of the specifications of our proposed thermal camera design (Table 4) with the state-of-the-art research designs (Table 3) shows that the following four main observations can be made: (1) Our f-number is slightly larger than the typically reported values, which are within the 0.8–1.1 range. (2) Our FOV competes with the state of the art. Only the LWIR earth sensor shows a larger FOV, but it features a lower MTF than our design. (3) Our total optical length is shorter than the lengths of the typical designs. Only the “low-cost thermal” design shows a shorter length, but it has a lower MTF and a smaller FOV. (4) Our design shows excellent image quality, surpassing the state of the art. Only optical designs featuring a smaller FOV and/or longer optical lengths can compete with our presented image contrast.

**Figure 13:**
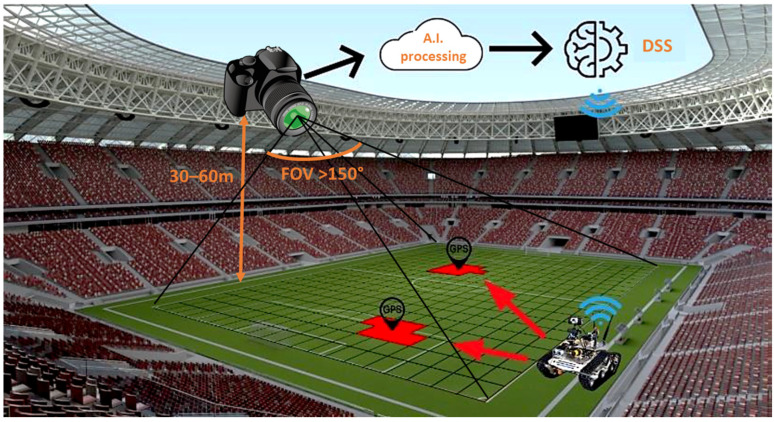
Sustainable and efficient turfgrass monitoring by integration of the multichannel camera within a fully robotized system, in combination with deep-learning processing and a decision-support system, a weather station, a mobile-sensing platform and an agriculture robot.

In the future, we will aim to integrate the developed multispectral camera design into a fully robotized system, GeenGo, enabling the automated acquisition of multispectral images while enabling in-software processing based on machine learning (Figure 13). More precisely, the cameras will be combined with a weather station, deep-learning image processing, a mobile sensor platform that is based on the global positioning system (GPS) and an agrobot (agriculture robot). The combined system will allow greenkeepers to know the precise health of the plant, providing insights into nutritional deficiencies and water stress. In addition, if a deficit or excess is detected, a warning will be sent to the farmer’s mobile phone, offering the choice of human inspection or an accurate treatment by the agriculture robot, paving the way toward the efficient management of the crop, working in a preventive way and saving time and costs.

Future work will include practical integration but also image processing, including both image correction and deep learning. Image processing that includes different spectral imaging channels has previously been illustrated by Dandrifosse et al., who considered combining visible, NIR and thermal images and indicated that image fusion was a promising processing technique [51]. The image fusion was demonstrated specifically on a small FOV design (<40° full FOV) and included short image distances, but it also offered a useful approach for our presented design. Thanks to our WFOV design, barrel distortion was identified as a main aberration. This distortion can be corrected by using standardized techniques, while orthorectification can be applied to obtain a planimetrically correct image, removing the effects of image perspective (tilt) and relief.

The thermal images and measured temperatures require a calibration with respect to environmental factors such as varying air temperature, wind speed and irradiance [52]. Therefore, the data of the weather station will provide indispensable input within this evaluation. Finally, postprocessing that is based on deep learning and that uses a trained neural network will be in place to gain insights into the vegetation indices, including those for weeds, stress and pathogen recognition, while monitoring the water and nutrient requirements.

The outcome of signal processing will drive a decision-support system, which can trigger a management alarm for the farmer or initiate an agrobot, enabling high-precision treatments of the turfgrass area. The agrobot will be guided to the correct location by using a virtual georeferenced grid. The outdoor mounting can be achieved by using available outdoor camera cases, such as those used in security cameras.

## 5. Conclusions

We presented a novel wide-field-of-view multispectral camera system featuring five imaging channels, covering the visible, near-infrared and thermal wavelengths bands. This system was suitable for integrating it into the lighting fixtures and offered the continuous monitoring of a wide inspection area thanks to its wide field of view, ≥ 164°. Each of the five imaging channels was optimized toward having a field of view, a spectral sensing region and high image quality. Excellent image quality was observed, competing with the state of the art while being implementable in a compact design. The proposed multisensor technology paves the way toward sustainable turfgrass monitoring to limit the use of chemical treatments while optimizing water management.

## Figures and Tables

**Table 4:** Overview of the design specifications of our proposed five-channel camera design.

Camera Channel	Wavelength Range (nm)	Full FOV (Degree)	Effective Focal Length (mm)	f-Number	Total Optical Length (mm)	MTF Value at a Frequency (lp/mm)	Detector Format (mm)	Pixel Size (µm)
Visible—NIR channel 1	648–750 nm	179°	1.58	2.8	29.8	≥0.5 @ 72	5.70 × 4.28	2.2
Visible—NIR channel 2	556–634 nm	179°	1.58	2.8	29.8	≥0.5 @ 72	5.70 × 4.28	2.2
Visible—NIR channel 3	641–697 nm + 823–900 nm	179°	1.58	2.8	29.8	≥0.5 @ 72	5.70 × 4.28	2.2
Visible—NIR channel 4	400–723 nm	179°	1.58	2.8	29.8	≥0.5 @ 72	5.70 × 4.28	2.2
Thermal	8–14 µm	164°	2.6	1.4	43.7	≥0.5 @ 27	7.68 × 5.76	12

## Data Availability

The data presented in this study are available on request from the corresponding author.
